# Biochemical and structural analyses reveal that the tumor suppressor neurofibromin (NF1) forms a high-affinity dimer

**DOI:** 10.1074/jbc.RA119.010934

**Published:** 2019-12-13

**Authors:** Mukul Sherekar, Sae-Won Han, Rodolfo Ghirlando, Simon Messing, Matthew Drew, Dana Rabara, Timothy Waybright, Puneet Juneja, Hugh O'Neill, Christopher B. Stanley, Debsindhu Bhowmik, Arvind Ramanathan, Sriram Subramaniam, Dwight V. Nissley, William Gillette, Frank McCormick, Dominic Esposito

**Affiliations:** ‡ NCI RAS Initiative, Cancer Research Technology Program, Frederick National Laboratory for Cancer Research, Frederick, Maryland 21702; § Helen Diller Comprehensive Cancer Center, University of California San Francisco, San Francisco, California 94158; ¶ Department of Internal Medicine, Seoul National University Hospital, Seoul 03080, Republic of Korea; ‖ Laboratory of Molecular Biology, NIDDK, National Institutes of Health, Bethesda, Maryland 20892; ** Robert P. Apkarian Integrated Electron Microscopy Core, Emory University, Atlanta, Georgia 30322; ‡‡ Oak Ridge National Laboratory, Oak Ridge, Tennessee 37830; §§ Argonne National Laboratory, Lemont, Illinois 60439; ¶¶ Frederick National Laboratory for Cancer Research, Frederick, Maryland 21702; ‖‖ Department of Biochemistry, Life Sciences Center, University of British Columbia, Vancouver, British Columbia V6T1Z3, Canada

**Keywords:** GTPase-activating protein (GAP), dimerization, Ras protein, GTPase Kras (KRAS), cell signaling, cancer, mitogen-activated protein kinase pathway, neurofibromin, NF1

## Abstract

Neurofibromin is a tumor suppressor encoded by the *NF1* gene, which is mutated in Rasopathy disease neurofibromatosis type I. Defects in *NF1* lead to aberrant signaling through the RAS–mitogen-activated protein kinase pathway due to disruption of the neurofibromin GTPase-activating function on RAS family small GTPases. Very little is known about the function of most of the neurofibromin protein; to date, biochemical and structural data exist only for its GAP domain and a region containing a Sec-PH motif. To better understand the role of this large protein, here we carried out a series of biochemical and biophysical experiments, including size-exclusion chromatography–multiangle light scattering (SEC-MALS), small-angle X-ray and neutron scattering, and analytical ultracentrifugation, indicating that full-length neurofibromin forms a high-affinity dimer. We observed that neurofibromin dimerization also occurs in human cells and likely has biological and clinical implications. Analysis of purified full-length and truncated neurofibromin variants by negative-stain EM revealed the overall architecture of the dimer and predicted the potential interactions that contribute to the dimer interface. We could reconstitute structures resembling high-affinity full-length dimers by mixing N- and C-terminal protein domains *in vitro*. The reconstituted neurofibromin was capable of GTPase activation *in vitro*, and co-expression of the two domains in human cells effectively recapitulated the activity of full-length neurofibromin. Taken together, these results suggest how neurofibromin dimers might form and be stabilized within the cell.

## Introduction

Neurofibromatosis type 1, a disease characterized by benign neurofibromas and malignant tumors of the nervous system, is caused by mutations in the 300-kb tumor suppressor gene, *NF1*, that encodes the protein neurofibromin (1). NF1 is also mutated in various types of cancer, including melanoma (2), leukemia (3), and lung (4). Neurofibromin is a large, multidomain protein with its best-described activity being the down-regulation of the mitogen-activated protein kinase pathway through its GTPase-activating protein (GAP)^4^ domain (5, 6), which stimulates RAS GTPase activity to shut off signaling. Given that there are no mutational hot spots in *NF1* (with over 2000 mutations reported in the Human Gene Mutation Database) (7) and that the phenotypes reported for mutations are diverse (8), it is possible that other *NF1* domains have a role in human disease. Thus, increasing our knowledge of neurofibromin structural biology is an important foundation for improving human health.

Unsurprisingly for such a large multidomain protein, neurofibromin is reported to interact with several proteins: SPRED family proteins (9), tubulin (10), kinesin-1 (11), protein kinase A (12), protein kinase C (13), caveolin (14), and amyloid precursor protein (15). Accordingly, of the several domains identified in neurofibromin by homology (cysteine/serine-rich domain, tubulin-binding domain (TBD), GAP-related domain (GRD), Sec-PH domain, and focal adhesion kinase-interacting domain), several are known to be protein/protein interaction domains. To date, detailed structural information on neurofibromin is limited to high-resolution crystal structures of the GAP domain (16), the Sec-PH domain (17, 18), and the ternary complex between KRAS4b, the EVH1 domain of SPRED1, and the GRD from NF1.^5^ No detailed information exists about the structure or function of the remaining 80% of the protein.

We report here the production of purified full-length neurofibromin and a number of fragments representing various domains of the protein. We demonstrate that neurofibromin exists as a high-affinity dimer *in vitro* and in cells. From EM experiments on full-length neurofibromin and domains, we propose a possible model for organization of the neurofibromin dimer and identify regions important for dimer formation. Collectively, this work provides a foundation for investigating mechanisms underlying the role of neurofibromin in the diverse collection of human diseases associated with this protein.

## Results

### Neurofibromin forms a high-affinity dimer in vitro

**Figure 1.**
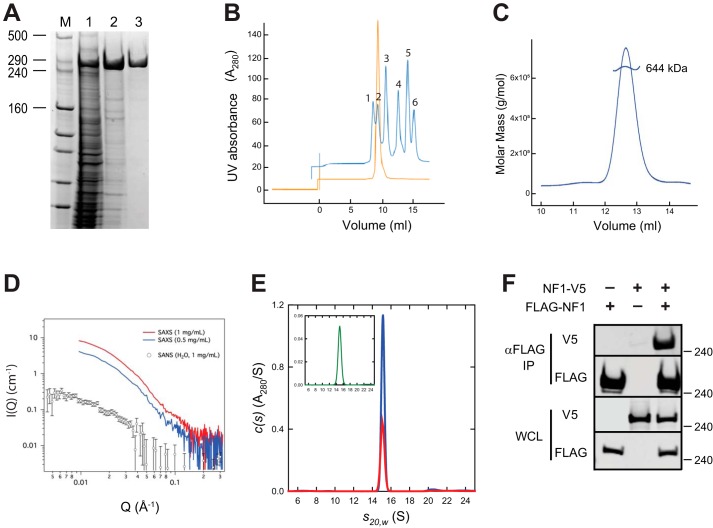
**Full-length neurofibromin is a high-affinity dimer.** *A*, SDS-PAGE analysis of purified full-length neurofibromin. *Lanes* are molecular mass markers (*M*) with the sizes of relevant bands shown in kDa, clarified extract from the insect cell expression (*lane 1*), elution pool from IMAC chromatography (*lane 2*), and purified protein after size-exclusion chromatography (*lane 3*). *B*, analytical SEC trace of purified neurofibromin (*orange*) compared with molecular weight standards (*blue*). Standards used were blue dextran (*peak 1*), thyroglobulin (*peak 2*, 669 kDa), ferritin (*peak 3*, 440 kDa), aldolase (*peak 4*, 158 kDa), conalbumin (*peak 5*, 75 kDa), and ovalbumin (*peak 6*, 43 kDa). *C*, SEC-MALS analysis of full-length neurofibromin. *D*, SAXS/SANS analysis of full-length neurofibromin. *Red* (1 mg/ml) and *blue* (0.5 mg/ml) *lines* are SAXS data from runs with two different concentrations of neurofibromin, and *open circles* represent SANS data using 1 mg/ml neurofibromin. *E*, sedimentation velocity absorbance *c*(*s*) profiles for NF1 at 0.6 μm (*red*) and 1.2 μm (*blue*) based on data collected at 280 nm. The *inset* shows the corresponding *c*(*s*) profile for NF1 at 25 nm (*green*) based on absorbance data collected at 230 nm. *F*, Western blotting of immunoprecipitation of differentially epitope-tagged NF1 proteins from HEK293 cells. The *top two gel sections* contain lysate purified with anti-FLAG antibodies. The *bottom two sections* contain WCL. In both cases, the samples are probed with antibodies to the FLAG or V5 epitopes as noted. Molecular mass of standards is noted on the *right* in kilodaltons.

Purified full-length human neurofibromin produced in baculovirus-infected insect cells migrated on SDS-PAGE at a molar mass close to its predicted size of 317 kDa (Fig. 1*A*). However, the purified protein eluted from a size-exclusion column at a position equivalent to the 669-kDa thyroglobulin standard (Fig. 1*B*) suggesting that purified neurofibromin existed as a dimer. To confirm this, additional measurements were made including size-exclusion chromatography–multiangle light scattering (SEC-MALS), which calculated a molar mass of 644 kDa (Fig. 1*C*), again consistent with a dimeric form of the protein. Additionally, we used small-angle X-ray scattering (SAXS) and small-angle neutron scattering (SANS) to investigate the structure and oligomeric state of neurofibromin in solution (Fig. 1*D*). SAXS was performed at two neurofibromin concentrations (0.5 and 1.0 mg/ml) with both yielding similar results, and SANS was also used to obtain lower *Q* data (<0.01 Å^−1^). The protein molar masses predicted from SAXS and SANS were in agreement, consistent with a dimeric architecture for neurofibromin (Table S1). Finally, analytical ultracentrifugation was used to conclusively characterize the oligomeric state of full-length neurofibromin across a range of concentrations. Analysis of sedimentation velocity and sedimentation equilibrium experiments at various concentrations ranging from 1.2 μm to 25 nm showed the presence of a single dominant species across the entire concentration range, which sedimented at 15.04 S with an estimated molar mass of 610 kDa (Fig. 1*E*) and a best-fit molar mass of 620 ± 70 kDa (Fig. S1). At a concentration of 25 nm, the absence of any secondary peak sedimenting at a monomeric size puts an upper limit on the affinity of the dimer at just below 1 nm. At that affinity, one would expect to see 10% of the protein in the monomeric state, which would be readily detectable in the sedimentation experiment. Overall, these data strongly supported the conclusion that full-length neurofibromin forms a high-affinity (*K*_dim_ <1 nm) dimer *in vitro*. To confirm this interaction *in vivo*, differentially epitope-tagged NF1 constructs (FLAG and V5 epitopes) were co-transfected into HEK293T cells. Antibodies against the FLAG epitope were used to immunoprecipitate (IP) FLAG-tagged neurofibromin, and precipitated samples were probed with anti-FLAG and anti-V5 antibodies (Fig. 1*F*). V5-tagged neurofibromin was readily detected in the FLAG IP sample, providing evidence of an interaction between full-length neurofibromin proteins in cells, consistent with dimerization.

### Negative stain EM shows the dimeric structure of neurofibromin

**Figure 2.**
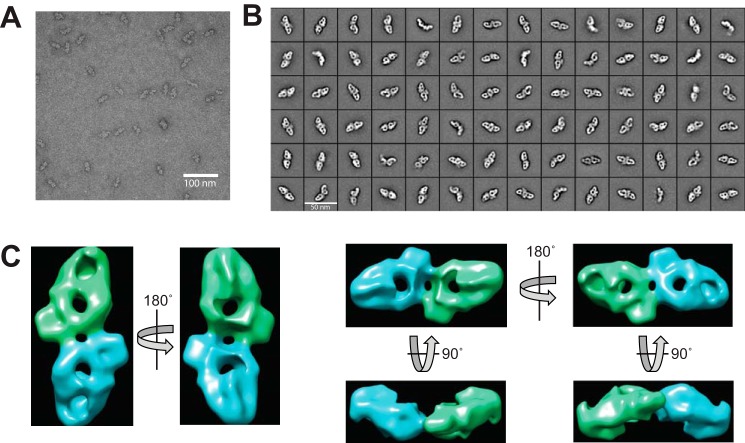
**Negative-stain EM of full-length neurofibromin.** *A*, representative transmission electron micrograph of full-length neurofibromin protein. *Scale bar* (100 nm) is shown for comparison. *B*, representative 2D class averages obtained from 41,951 particles. Each *box* shown is 50 × 50 nm. *C*, 20-Å density map of full-length neurofibromin generated from 294 class averages shown in multiple orientations. Proposed protomers are indicated with *green* and *blue coloring*.

Projection images of negatively-stained neurofibromin obtained by transmission EM analysis allowed visualization of the neurofibromin particle as a pseudo-symmetric dimer (Fig. 2, *A* and *B*) in which the two NF1 protomers appear as oblong lobes connected by an interface region. Inspection of the 2D class averages suggest the dimeric arrangement is conformationally heterogeneous, with indications of variability both in the quaternary structure and in the individual protomers. 3D reconstruction of the structure at ∼20 Å resolution (Fig. 2*C*, individual protomers shown in different colors) using the dominant 2D classes allows visualization of the densities corresponding to the two NF1 protomers. The complex has overall dimensions of ∼32 nm along the long axis of the dimer. Back projections from this model using EMAN2 to generate 2D class averages were used to validate the accuracy of the model (Fig. S2). 3D variability analysis (Fig. S3) highlights the unreliable parts of the reconstruction corresponding to regions of diffuse density seen in some 2D class averages. These data support the notion of some level of conformational flexibility in portions of the neurofibromin dimer. A theoretical scattering curve generated from the reconstructed 3D model (Fig. S4, *black line*) shows excellent agreement with the experimental SAXS data. The SANS prediction of a maximum dimension (*D*_max_) in solution of ∼30 nm is in reasonable agreement with predictions from the negative stain data and suggests that the reconstruction may be slightly larger than the actual solution size due to flattening or dehydration effects during EM grid preparation.

### N-terminal domains of neurofibromin are incapable of dimerization

**Figure 3.**
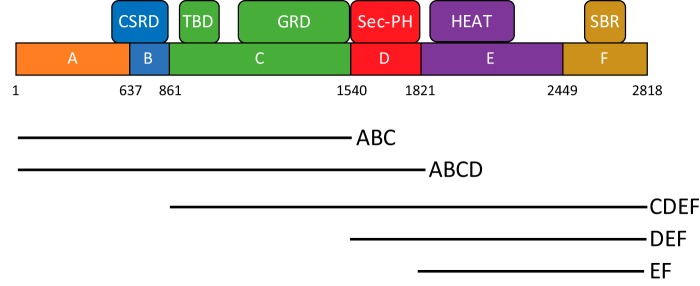
**Neurofibromin fragments and domain architecture.** Schematic of the regions utilized for production of neurofibromin proteins in this study. Domains have amino acid boundaries as noted and are designated by *letters A–F*. Corresponding regions of interest identified from the literature are noted *above*. Abbreviations used are as follows: *CSRD*, cysteine–serine-rich domain; *SBR*, syndecan-binding region.

**Figure 4.**
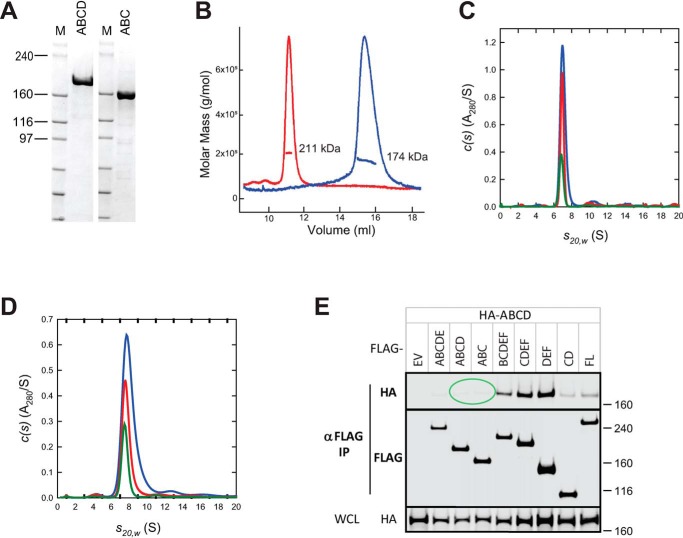
**N-terminal domains of neurofibromin are monomeric.** *A*, SDS-polyacrylamide gel of purified ABC and ABCD proteins. Size of molecular mass markers are noted in kilodaltons. *B*, SEC-MALS analysis of ABC (*blue*) and ABCD (*red*) fragments. *C*, sedimentation velocity absorbance *c*(*s*) profiles for the ABC protein at 1.1 μm (*green*), 2.2 μm (*red*), and 4.5 μm (*blue*) based on data collected at 280 nm. *D*, sedimentation velocity absorbance *c*(*s*) profiles for the ABCD protein at 1.1 μm (*green*), 2.1 μm (*red*), and 4.2 μm (*blue*) based on data collected at 280 nm. *E*, Western blotting of immunoprecipitation of differentially epitope-tagged NF1 proteins in HEK293 cells. In this figure, HA-ABCD protein was co-IPed with FLAG-tagged domains as noted. The *top two gel sections* are lysates purified with anti-FLAG antibodies and probed with antibodies to the HA or FLAG epitopes. The *bottom section* contains WCL probed with anti-HA antibodies. Molecular mass of standards is noted on the *right* in kilodaltons. *Circled regions* are discussed in more detail in the text. *EV*, empty vector control; *FL*, full-length NF1.

To investigate the elements of neurofibromin that might be required for dimerization, we divided the protein into a series of domains (Fig. 3, *A–F*) based on bioinformatic analysis, including phylogenetic homology, intron/exon boundaries, secondary structure prediction, and surface entropy prediction. DNA constructs representing the various combinations of domains were generated that corresponded to 21 different protein fragments. A subset of these proteins was purified and used for the experiments discussed here (Table 1). N-terminal ABC and ABCD fragments were purified at high yield and appeared well-behaved based on size-exclusion chromatography, differential scanning fluorimetry, and light scattering, and they were utilized for biochemical analysis. Under reducing conditions, both ABC and ABCD migrated on SDS-PAGE (Fig. 4*A*) near their expected molar masses (173 and 206 kDa, respectively). SEC-MALS produced sharp peaks corresponding to molar masses of 174 and 211 kDa, respectively (Fig. 4*B*), suggesting that these proteins exist in solution as monomers. This result was confirmed using analytical ultracentrifugation, in which sedimentation velocity of the ABC domain at multiple concentrations (Fig. 4*C*) showed the presence of a species sedimenting at 6.88 S with an estimated molar mass of 165 kDa, consistent with the size of an ABC monomer. Similar observations were made with ABCD at 1.1 μm and showed the presence of a species at 7.58 S with an estimated molar mass of 190 kDa (Fig. 4*D*), confirming that the ABCD domain is also monomeric. The smaller than expected mass can be attributed to the weak self-association observed as the concentration was raised from 1.1 to 4.4 μm. SAXS data also confirmed that the ABCD domain was monomeric in solution (Table S1 and Fig. S5). Transmission EM studies of the ABC and ABCD proteins showed elongated particles of a monomeric size that could not be consistently class-averaged, suggesting a flexible structure (Fig. S6, *A* and *B*). Similar domain boundaries were used to engineer constructs for expression in HEK293T cells in which endogenous NF1 was deleted on both alleles. Differentially epitope-labeled ABC and ABCD proteins did not co-immunoprecipitate (co-IP), confirming the lack of interaction of these monomeric domains *in vivo* (Fig. 4*E*, *green circled region*).

**Table 1:** **Neurofibromin proteins used in this study** Cited are the amino acid regions present in purified protein domains, along with the predicted isoelectric point (pI) and the predicted molecular mass of each final protein. Also shown is the construct from which the proteins were purified and the host organism used for protein expression. In the case of His_6_-MBP fusion proteins, the final purified proteins were cleaved from the MBP solubility tag, and the full-length His_6_-tagged neurofibromin remained tagged after purification. Constructs C* and C*D have altered start sites within the C domain compared with other C domain–containing proteins.

Domain	Amino acids	pI	Mass (*kDa*)	Exp. host	Construct
Full length	2–2818	6.9	317	Tni-FNL	His_6_-tev-NF1
ABC	2–1540	6.4	173	Tni-FNL	His_6_-MBP-tev-NF1(2–1540)
ABCD	2–1820	6.5	206	Tni-FNL	His_6_-MBP-tev-NF1(2–1820)
CDEF	861–2818	6.9	220	Tni-FNL	His_6_-MBP-tev-NF1(861–2818)
DEF	1541–2818	8.0	144	Tni-FNL	His_6_-MBP-tev-NF1(1541–2818)
EF	1821–2818	8.1	111	Tni-FNL	His_6_-MBP-tev-NF1(1821–2818)
C*D	1085–1816	6.4	83	Tni-FNL	His_6_-MBP-tev-NF1(1085–1816)
D	1541–1820	7.3	32	Tni-FNL	His_6_-MBP-tev-NF1(1541–1820)
C*	1085–1530	6.0	50	Tni-FNL	His_6_-MBP-tev-NF1(1085–1530)
GRD	1172–1530	6.0	41	*E. coli*	His_6_-MBP-tev-NF1(1172–1530)
GRD	1198–1530	6.4	38	*E. coli*	His_6_-MBP-tev-NF1(1198–1530)
GRD	1203–1530	6.6	38	*E. coli*	His_6_-MBP-tev-NF1(1203–1530)

### C-terminal domains of neurofibromin are capable of dimerization

**Figure 5.**
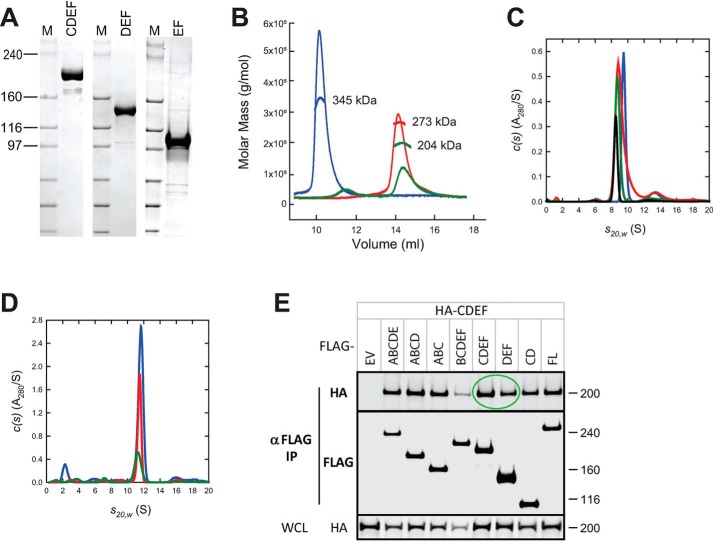
**C-terminal domains of neurofibromin are capable of dimerization.** *A*, SDS-polyacrylamide gel of purified CDEF, DEF, and EF proteins. Size of molecular mass markers are noted in kilodaltons. *B*, SEC-MALS analysis of CDEF (*blue*), DEF (*red*), and EF (*green*) proteins. *C*, sedimentation velocity absorbance *c*(*s*) profiles for the DEF protein at 2.1 μm (*green*), 4.2 μm (*red*), and 8.8 μm (*blue*) based on data collected at 280 nm. *D*, sedimentation velocity absorbance *c*(*s*) profiles for the CDEF protein at 1.2 μm (*black*), 2.6 μm (*green*), 5.2 μm (*red*), and 10.5 μm (*blue*) based on data collected at 280 nm. Data at the highest concentration was collected using a 3-mm pathlength cell, and standard 12-mm cells were used for the lower concentrations. *E*, Western blotting of co-immunoprecipitation of differentially epitope-tagged NF1 proteins in HEK293 cells. In this figure, HA-CDEF protein was co-IPed with FLAG-tagged domains as noted. The *top two gel sections* are lysates purified with anti-FLAG antibodies and probed with antibodies to the HA or FLAG epitopes. The *bottom section* contains WCL probed with anti-HA antibodies. Molecular mass of standards is noted on the *right* in kilodaltons. *Circled regions* are discussed in more detail in the text. *EV*, empty vector control; *FL*, full-length NF1.

As the N-terminal end of neurofibromin did not appear capable of forming dimers, we next examined the C-terminal end of the protein using fragments CDEF, DEF, and EF. All three polypeptides could be produced and purified at high yield (Fig. 5*A*) and migrated near their expected molar masses on SDS-PAGE (220, 144, and 111 kDa, respectively). SEC-MALS data, however, showed anomalous migration of these proteins, yielding molar masses of 345 kDa (CDEF), 273 kDa (DEF), and 204 kDa (EF), suggesting that these proteins were capable of self-association into higher-order structures (Fig. 5*B*). Sedimentation data for DEF collected at 1.1 μm showed the presence of a species at 8.52 S with an estimated mass of 170 kDa. When the concentration was increased to 10.5 μm, a species was observed at 9.56 S (Fig. 5*C*). The larger than monomer mass observed for the 8.52 S species at 1.1 μm, the observation of intermediate sedimentation coefficient species at concentrations between 1.1 and 10.5 μm, and the observation of a best-fit frictional coefficient of less than unity at 10.5 μm support a weak self-association with fast exchange (19). Sedimentation experiments on CDEF at 8.8 μm showed the presence of a species at 11.65 S with an estimated molar mass of 300 kDa (Fig. 5*D*). These data suggest that the CDEF domain is dimeric and the smaller than expected mass can be attributed to the self-association observed as the concentration is raised from 2.2 to 8.8 μm. SAXS data on CDEF at 15 μm also confirmed that this protein was entirely dimeric in solution at a high concentration (Table S1 and Fig. S5). The oligomerization of this domain is strongly concentration-dependent; based on SAXS and analytical ultracentrifugation data, CDEF exists as a dimer at concentrations above 150 nm. At the lower concentrations used for negative stain EM analysis (45 nm), a mixed population of monomers and dimers was observed (Fig. S6*C*).

Finally, using HEK293 cells lacking endogenous NF1, we were able to show that the CDEF and DEF domains of differentially epitope-tagged neurofibromin did co-IP (Fig. 5*E*, *green circled region*), confirming the potential for dimerization in cells similar to that observed with purified proteins.

### Equimolar mixtures of N- and C-terminal domains of neurofibromin can reconstitute the full-length neurofibromin dimer

**Figure 6.**
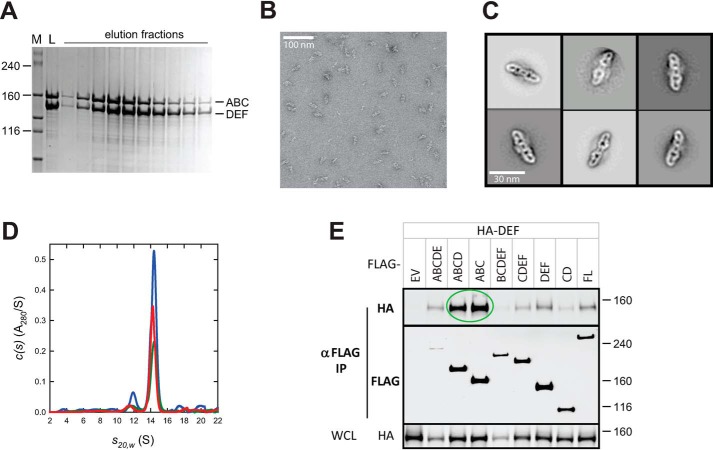
**Reconstitution of full-length neurofibromin dimers from ABC and DEF proteins.** *A*, SDS-PAGE analysis of SEC of a equimolar mixture of ABC and DEF proteins. Size of molecular mass markers in *lane M* are noted in kilodaltons. *Lane L* represents the loaded material, and additional lanes are elution fractions across the column. *B*, transmission electron micrograph of reconstituted neurofibromin dimers from the ABC/DEF mixture. *Scale bar* (100 nm) is noted. *C*, representative 2D class averages from particles selected from multiple transmission electron microscopic micrographs. *Scale bar* (30 nm) is noted. *D*, sedimentation velocity absorbance *c*(*s*) profiles for equimolar mixtures of the N-terminal ABC and C-terminal DEF fragments of NF1 at 1.0 μm (*green*), 2.0 μm (*red*), and 4.5 μm (*blue*) based on data collected at 280 nm. Data at the highest concentration were collected using a 3-mm pathlength cell; standard 12 mm cells were used for the lower concentrations. *E*, Western blotting of co-immunoprecipitation of differentially epitope-tagged NF1 proteins in HEK293 cells. In this figure, HA-DEF protein was co-IPed with FLAG-tagged domains as noted. The *top two gel sections* are lysates purified with anti-FLAG antibodies and probed with antibodies to the HA or FLAG epitopes. The *bottom section* contains WCL probed with anti-HA antibodies. Molecular mass of standards are indicated on the *right* in kilodaltons. *Circled regions* are discussed in more detail in the text. *EV*, empty vector control; *FL*, full-length NF1.

The ability of CDEF to self-associate but not form dimers at concentrations observed for the full-length protein suggests that the subdomains may lack critical intramolecular or intermolecular contacts that stabilize the dimer. To test this hypothesis, purified ABC and DEF proteins were mixed in an equimolar concentration and subjected to size-exclusion chromatography. Surprisingly, the major peak of protein eluted off of the SEC column at a size corresponding to 600 kDa, similar to that observed with full-length neurofibromin dimers. SDS-PAGE analysis confirmed that both subdomains were present in this complex at what appeared to be stoichiometric levels (Fig. 6*A*). Sedimentation velocity experiments were carried out on the purified complex of ABC and DEF. Complexes made from an equimolar mixture of ABC and DEF in the 1.0–4.5 μm concentration range showed the presence of a species at 14.32 S with an estimated mass of 580 ± 70 kDa (Fig. 6*D*). We observed similar reconstitution results using ABCD and EF proteins, and sedimentation studies of these mixtures in the 1.0 to 5.5 μm concentration range also showed the presence of a species at 14.26 S with an estimated mass of 580 ± 95 kDa (data not shown). Molar masses represent the average from all experiments, where the reconstituted dimer represents ∼65 and 85% of the sedimenting signal for ABC/DEF and ABCD/EF, respectively. Sedimentation data over a range of concentrations suggest that these complexes have similar dimerization affinities (<5 nm) as the full-length neurofibromin dimers. Electron microscopic images and the resulting 2D class averages show the presence of structures in the reconstituted complexes that are similar to those observed with full-length neurofibromin dimers (Fig. 6, *B* and *C*). A low-resolution model was generated from these data, and the resulting 3D image also is strikingly similar to the model of full-length neurofibromin (Fig. S7). These results suggest that a mixture of monomeric N-terminal fragments such as ABC with C-terminal fragments capable of dimerization such as DEF can recapitulate full-length dimeric neurofibromin structures with similar high affinity. Co-IP data in HEK293 cells (Fig. 6*E*) also confirm that ABC and DEF proteins are capable of interactions in cells that permit pulldown of differentially tagged proteins; here, both N-terminal fragments (ABC and ABCD) co-IP with the C-terminal DEF fragment as predicted by the *in vitro* protein reconstitution data, as also shown in Fig. 5*E* for the CDEF fragment.

### Identification of regions responsible for neurofibromin dimerization

To identify the regions responsible for dimerization, we investigated interactions between the C-terminal EF domain and regions upstream in neurofibromin. We measured interactions using SEC by mixing the EF domain (1821–2818) with multiple domains in the C/D regions of neurofibromin. Although both the C*D domain (1085–1816, Fig. 7*A*) and the C* domain (1085–1530, Fig. 7*B*) co-eluted, indicating complex formation, the D domain (1541–1820, Fig. 7*C*) did not. This suggested that the interacting domain was in the C domain, which contains the tubulin-binding domain (TBD, 1085–1194) and the GAP-related domain (GRD, 1198–1530). Using additional NF1 GRD-region proteins, we were able to further delimit the region between amino acids 1085–1172 as essential for interaction with the EF domain (Fig. S8). We were also able to confirm that the same region was essential for interaction with the DEF domain (Fig. S9), suggesting that the TBD is the critical C-domain component for neurofibromin multimerization.

**Figure 7.**
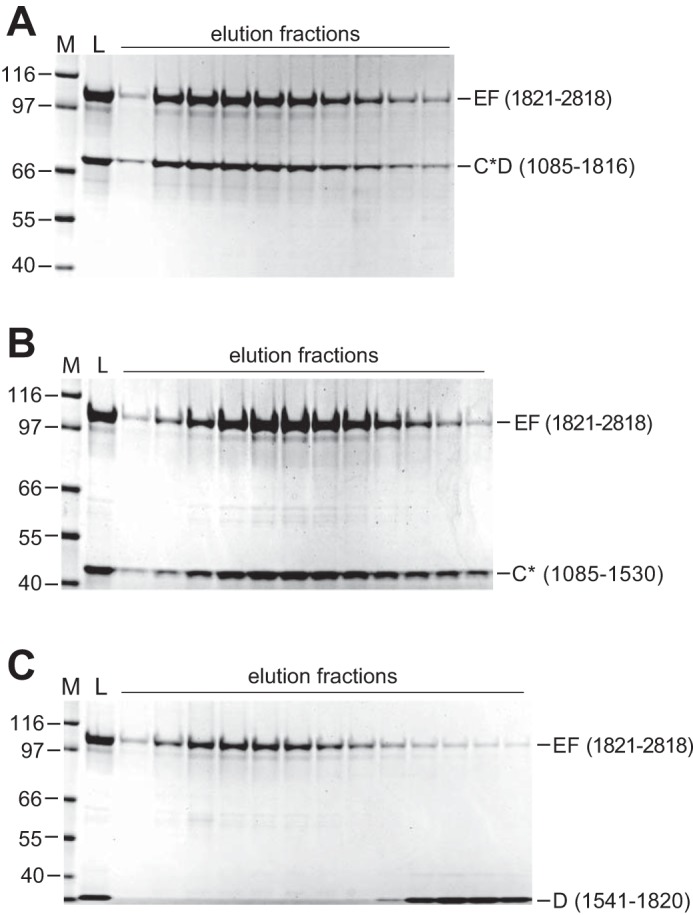
**Identification of the interaction between the neurofibromin C and EF domains.** SDS-PAGE analysis of SEC of equimolar mixtures of NF1 domains with EF(1821–2818). In each panel, the size of molecular mass markers in *lane M* is noted in kilodaltons. *Lane L* represents the loaded material, and additional lanes are elution fractions across the column. Neurofibromin fragments mixed with the EF domain in each panel were as follows: *A*, 1085–1816; *B*, 1085–1530; *C*, 1541–1820.

### Measurement of neurofibromin domain GTPase stimulation activity

**Figure 8.**
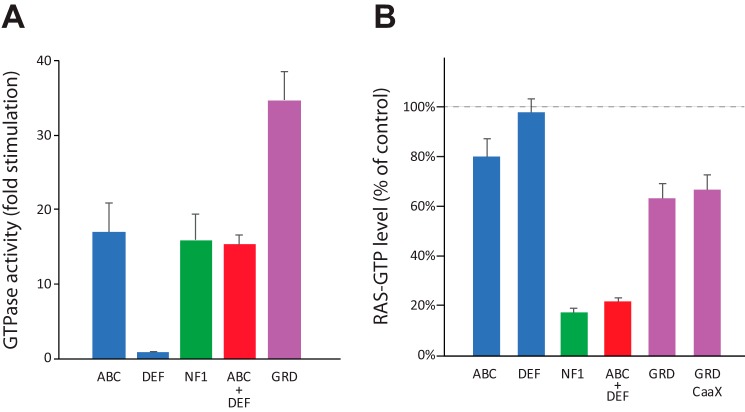
**RAS GTPase stimulation by neurofibromin domains.** *A*, phosphate sensor assay using purified neurofibromin proteins. The level of stimulation of basal RAS GTPase activity is shown for four different neurofibromin proteins and an equimolar mixture of ABC and DEF proteins. Data shown are the averages of two separate assays, each performed in triplicate, with standard deviations shown with *error bars. B*, RAS–GTP ELISA carried out in HEK293 cells lacking endogenous NF1. The plotted data are measurements of RAS–GTP levels after EGF stimulation of serum-starved cells that were transfected with various NF1 constructs. Data are percentages of a control experiment with GFP and are averages of three or four replicate wells for each sample. Standard deviations are noted with *error bars*.

To assess the biological relevance of some of the neurofibromin domains studied in this work, assays were used to measure the level of GAP activity in cells using purified proteins. We first examined the ability of purified neurofibromin and domains to stimulate the intrinsic GTP hydrolysis activity of RAS using an assay that monitors release of P_i_ in a single-turnover reaction (Fig. 8*A*). As expected, proteins containing the C domain, in which the GRD resides, showed significant stimulation of GAP activity. At identical concentrations of protein, the monomeric ABC protein stimulated GTPase activity to a similar extent as full-length neurofibromin (Fig. 8*A*, *green bar*) and the reconstituted complex of ABC + DEF (Fig. 8*A*, red bar), suggesting that the multimeric state of the protein harboring the GRD domain was not correlated to activity *in vitro*. Interestingly, the isolated GRD domain (1198–1530) consistently showed higher levels of GAP activity compared with other NF1 domains, suggesting that some inhibition of activity in the larger domains may be reversed once N-terminal regions of the protein are removed. As expected, the DEF fragment, which lacks the GRD domain, shows no stimulation of activity over the intrinsic hydrolysis rate of RAS.

To assess activity of these proteins in cells, constructs containing epitope-tagged neurofibromin domains were transfected into serum-starved HEK293 cells lacking endogenous NF1, and levels of RAS–GTP after EGF stimulation were measured using an ELISA (Fig. 8*B*). In cells, although GRD domain constructs (with or without membrane localization) and ABC protein were able to slightly stimulate RAS GTPase levels by 20–30% over controls (DEF or GFP-transfected cells), full-length NF1 or a co-transfection of ABC and DEF fragments were able to produce considerably higher stimulation of RAS GTPase activity. Western blot analysis confirmed that all proteins were expressed at similar levels, ensuring that the measurement of GTPase stimulation was not impacted by differences in protein amounts.

## Discussion

The 2818 amino acid neurofibromin protein is involved in a variety of human diseases, including several sporadic cancers, as well as the common genetic disorder neurofibromatosis type I. Although the underlying mechanism of these diseases seems to involve defects in the GTPase-activating activity of neurofibromin, which regulates the RAS family of small GTPases and downstream mitogen-activated protein kinase pathways, this activity resides in a small portion of the protein. Very little is understood about the role of any of the remaining parts of neurofibromin. Also, missense mutations that lead to NF1 disease and NF1-driven cancers occur throughout the protein and are not localized specifically to the GAP-related domain (20). This argues for additional roles of these mutations in either neurofibromin activity or protein stability. To investigate some of these questions, we produced full-length human neurofibromin protein using an insect cell system. Size-exclusion chromatography experiments showed that this large protein appeared to exist in solution as a dimer. Additional biophysical measurements confirmed that the purified neurofibromin protein was dimeric and that the dimerization affinity was low enough that effectively all of the protein existed in solution as an obligate dimer at concentrations above 25 nm. We were able to confirm using cell-based methods that neurofibromin proteins expressed in HEK293 cells lacking endogenous NF1 also showed self-association, as differentially-tagged NF1 proteins could be used to co-IP neurofibromin dimers containing both tags. The dimeric organization of the protein was also verified in projection electron microscopic images and the resulting 3D reconstruction of purified neurofibromin. These findings are consistent with a report showing that Ira2, one of the two *Saccharomyces cerevisiae* paralogs of neurofibromin, could co-immunoprecipitate the other paralog, Ira1, as detected by MS (21). A published report noted that human neurofibromin eluted from size-exclusion chromatography during purification as an apparent dimer (22), and other studies have proposed that multimerization could potentially explain dominant-negative effects of heterozygous truncation mutations of NF1 in patients (23). A recent paper published during the preparation of this manuscript demonstrated that tagged murine neurofibromin could co-IP human neurofibromin in HEK293 cell lines, suggesting that these highly-homologous proteins from the two species (>98% identity) could form dimers as well (24).

To define the domain architecture of neurofibromin, we chose a directed bioinformatic approach in which the protein was broken down into six domains based on a series of protein-focused bioinformatic methods, and the various contiguous combinations of these domains were cloned and proteins generated using a baculovirus-based insect cell system. Previous attempts to generate neurofibromin fragments using a random library approach in *Escherichia coli* generated few useful proteins (25). However, in our study, with few exceptions, we were able to purify most of the neurofibromin fragment proteins using these domain boundaries. We focused on the well-behaved and readily purifiable N-terminal domains (ABC and ABCD) and C-terminal domains (CDEF, DEF, and EF) for additional studies. These proteins behaved as well-folded and structured domains in multiple analyses (ultracentrifugation, light scattering, and neutron scattering). Additionally, differential scanning fluorimetry was used to confirm the behavior of these important domains for further work; the thermal melting properties of these domains were consistent with those of well-folded proteins (Fig. S10). To alleviate concerns about the higher salt content in many of the buffers during size-exclusion chromatography, we repeated the experiments on several important domains at physiological salt concentrations (150 mm) with identical results.

N-terminal domains of neurofibromin (ABC and ABCD) produced monomeric proteins at all concentrations studied. Negative stain EM images of these domains suggested the presence of partially unfolded particles. In contrast, the C-terminal domains (CDEF, DEF, and EF) produced proteins that were capable of self-association in a concentration-dependent manner. In particular, the DEF and EF proteins showed very weak self-association that was evident only at the highest concentrations. However, the CDEF protein formed significant amounts of dimeric protein, and even at the low concentration used for EM analysis, a clear mixture of monomer and dimer populations was observed. This suggests that CDEF likely contains the major components responsible for the dimerization of the full-length protein. *In vivo* data using these domains confirmed these findings: ABC and ABCD proteins that were differentially tagged were unable to co-immunoprecipitate with each other *in vivo*, confirming that these proteins do not interact to form multimers. However, co-immunoprecipitation was observed with differentially-tagged CDEF and DEF proteins, suggesting again that the experiments with purified proteins recapitulate what is observed in cells.

The *in vivo* data also produced an unexpected result: the ABC protein *in vivo* was able to co-IP with the DEF protein. To investigate whether this phenomenon could be recapitulated *in vitro*, we mixed the purified ABC and DEF proteins and separated the mixture using size-exclusion chromatography. Surprisingly, a protein complex eluted from the SEC column at the size of a full-length neurofibromin dimer (600 kDa), suggesting that individual protomers of ABC and DEF were capable of recapitulating a structure similar to the full-length dimer. This complex behaved in analytical ultracentrifugation experiments exactly like full-length dimers, including a lack of concentration dependence suggesting a high-affinity interaction as observed for the full-length protein. Thermal melting data (Fig. S10) showed that the stability of full-length neurofibromin and the reconstituted complex of ABC + DEF were identical, with both proteins melting in a Gaussian distribution centered at 47.7 °C. Projection electron microscopic images from negatively-stained complexes were indistinguishable from those seen with the full-length protein, confirming that this mixture of domains could recapitulate the folding of full-length neurofibromin dimers. The number of available particles in these images was too low to permit a high-resolution model as was generated for the full-length protein; however, a lower resolution model was obtained and confirms that the reconstructed dimer is highly similar in shape and structure to the full-length dimer (Fig. S7). Similar reconstitution experiments were carried out with ABCD and EF proteins with identical results (data not shown). Notably, we did not observe any 1:1 complex of ABC + DEF proteins, suggesting that the protein/protein interface of this recapitulated dimer is similar to that of the WT dimer.

Based on these results, we focused on potential interactions with the EF domain. The E domain of neurofibromin contains a series of 12 predicted HEAT-like repeats. These repeats are commonly involved in protein/protein interactions, and they often form a complex solenoid structure incorporating a series of α-helices that can be used for additional interactions with partner proteins (26). From the reconstitution data, we tested the idea that an E domain solenoid might interact with a region upstream in neurofibromin, likely in the C or D regions. Both the purified C domain and CD domains were able to make complexes with EF, but the purified D domain was not, implicating the C domain (which contains the putative TBD and GRD) in the interaction. As several C domain constructs used for studies of the structure of the GAP domain of neurofibromin were available to test, we were able to show that the region of the C domain from 1085 to 1171 was required for complex formation with EF. We also confirmed that the TBD region of neurofibromin was able to form stable complexes with the DEF domain protein as well.

The neurofibromin TBD consists of three predicted amphipathic α-helices (1095–1113, 1134–1152, and 1173–1194), which we hypothesize might be capable of interacting with the E domain HEAT-like solenoid. This type of tight interaction involving a HEAT-like solenoid at a dimer interface has been observed with the adaptor protein mTOR. Cryo-EM studies have shown that the formation of high-affinity mTOR dimers is due to the hydrophobic interaction of a HEAT-like solenoid in the N-terminal region of mTOR with a set of three amphipathic helices in the core domain of a second monomer of mTOR (27). Like neurofibromin, the mTOR dimer has a high affinity (<10 nm) due to the strong interaction of these hydrophobic domains. The strength of these dimers is enhanced by the presence of multiple interactions, as each protomer contains both helical elements that lead to two large hydrophobic contact points along the dimer interface. We propose that the points of contact seen in the 3D reconstruction of neurofibromin likely represent C/E domain interactions, leading to a stable, high-affinity dimer. Although we do not have clear evidence for which portions of the neurofibromin 3D model represent which domains, we propose one possible configuration of the protein consistent with this putative C/E domain interaction (Fig. S11). This model predicts two structural roles for the ABC and DEF portions of NF1 that are supported by the observation of only full-length dimers in the ABC + DEF recapitulation experiment. We presume in this case that extensive intramolecular interactions of ABC and DEF drive the formation of the proper NF1 monomer conformation which then permits the efficient intermolecular interaction of C/E to form the high-affinity dimer.

Dimerization provides a potential explanation for the phenotypes observed in common NF1 disease mutations. Many of the mutations observed are heterozygous nonsense or frameshift mutations, which lead to premature truncation of neurofibromin. In most of these patients, there is still a single copy of WT NF1 present, and yet as seen in a recent study, the total amount of full-length neurofibromin in some of these patients is considerably less than the predicted 50% of WT levels (23). One explanation for this would be that the truncated forms of the protein are still capable of interactions with the WT protein, which could lead to enhanced proteosomal degradation of both the defective protein and the WT partner in the dimer. Effectively, these defective truncated proteins would serve as subunit poisons, still interacting with WT neurofibromin and interfering with the production of normal dimers. The biophysical techniques utilized in this work should serve as an effective means of testing these mutants to see whether their effects on WT neurofibromin can be recapitulated *in vitro*.

The biological consequence of dimerization of neurofibromin remains unclear. Interactions of purified RAS proteins with the NF1 GAP domain appear to involve monomers of both proteins, and the recently elucidated structure of KRAS–NF1 GAP–SPRED1 EVH also involves a 1:1:1 complex of monomeric proteins.^6^ To investigate the role of dimerization, we first validated the GTPase stimulation activity of various neurofibromin fragments. All proteins tested that contained the GRD region showed stimulation of RAS GTPase activity *in vitro*, with monomeric ABC, dimeric full-length, and the reconstituted ABC + DEF dimer all showing similar levels of stimulation. Because of the high affinity of the full-length neurofibromin dimer, it was impossible to measure the GAP activity of monomeric neurofibromin: at concentrations where any significant level of monomeric protein would exist, the GAP activity is too low to measure. The consistently higher activity of the isolated GRD domain does suggest that some feature of the larger neurofibromin proteins may reduce the GAP activity, perhaps by steric interference or simply as a result of altered protein stability. Nevertheless, these data add another example of the recapitulation of neurofibromin activity from the isolated ABC + DEF domains.

To expand on the *in vitro* data with purified proteins, we also looked at the effect of these domains in cells by transfecting various domain constructs into HEK293 cells lacking NF1. In this case, the data very clearly suggest an additional role other than direct GAP activity for the full-length neurofibromin protein in cells. As Fig. 8*B* shows, only full-length neurofibromin or the presumably reconstituted ABC + DEF dimer is capable of significant stimulation of RAS–GTP levels in these cells. Although a low level of stimulation (20–40%) is observed with any construct containing the GRD, the dramatic increase in stimulation with the full-length constructs clearly indicates a biological need for the full-length protein, which could represent the role of another portion of the NF1 protein outside of the GRD, or for the role of dimerization itself in regulation of neurofibromin activity. As the *in vitro* GAP activity of full-length dimers and monomeric GRD and ABC domains is similar, the dimerization may be playing a role in interactions with other proteins or in proper membrane localization or orientation of neurofibromin. Notably, this role cannot simply be movement of the GAP domain to the membrane, as the membrane-associated GRD–C*AAX* construct does not decrease the level of RAS–GTP significantly. Additional work will be needed to understand the full biological ramifications of neurofibromin dimerization and the potential other roles that neurofibromin may play in the activation of RAS GTPase.

## Experimental procedures

### Cloning of neurofibromin constructs

DNA constructs were generated for the various fragments and full-length neurofibromin using Gateway recombinational cloning (28). Entry clones were produced by PCR amplification of portions of the NF1 gene from Addgene construct 70423, which contains a codon-optimized version of human NF1 isoform 2 (NM_000267.3) to ensure stability of the plasmid in *E. coli*. 5′ sequences included Gateway *att*B1 sites followed by sequences for protease cleavage by tobacco etch virus (TEV) protease (ENLYFQ/G), whereas 3′ sequences contained stop codons and Gateway *att*B2 sites. PCR amplicons were cloned into pDonr255 and fully sequence-verified. Gateway LR recombination was used to generate final expression clones in baculovirus Destination vectors pDest-635 (pFastBac1 with N-terminal His_6_ tag) and pDest-636 (pFastBac1 with N-terminal His_6_-MBP tag) or *E. coli* Destination vector pDest-566 (N-terminal His6-MBP tag, Addgene catalog no. 11517). Final baculovirus expression clones were transformed into DH10Bac cells (Thermo Fisher Scientific), and bacmid clones were verified by PCR (29). *E. coli* expression clones were transformed into BL21(DE3) for expression. Plasmids for mammalian IP experiments were made by subcloning Entry clones into transient transfection Destination vectors (pCAN-FLAG-DEST, pCAN-HA-DEST, and pEF-DEST51) using Gateway LR recombination per the manufacturer's instructions. Plasmids for RAS–GTP ELISAs were generated by Multisite Gateway LR subcloning of domain and full-length neurofibromin Entry clones into vectors containing an elongation factor 1a (EF1a) promoter along with various N-terminal epitope tags (HA, V5, or FLAG).

### Protein production

Proteins were expressed in *E. coli* as described previously (30). For insect cell expression, baculoviruses were generated by transfection of bacmid DNA into Sf9 cells, as described previously (31). Titered viruses were used at a multiplicity of infection of 3 to infect Tni-FNL cells (32) that were grown at 21 °C for 72 h prior to harvest. Frozen insect cell pellets containing expressed neurofibromin proteins were thawed and homogenized at room temperature in 100 ml of lysis buffer (Table S2) per l of culture volume. Homogenized cell pellets were then lysed by passing through a Microfluidizer M-110EH (Microfluidics Corp.) twice at 7000 p.s.i. Lysate was clarified by ultracentrifugation at 104,600 × *g* for 40 min at 4 °C. Clarified lysate was filtered through 0.4-μm Whatman PES syringe filters (GE Lifesciences). All proteins were purified on NGC^TM^ chromatography systems (Bio-Rad). Most proteins were purified as described for proteins with TEV-cleavable His_6_-MBP N-terminal tags (33) with modifications to buffers as noted in Table S2. Lysis buffers were the base buffers used for the initial IMAC step. Full-length neurofibromin was produced with a construct containing an N-terminal His_6_ tag that was not removed during the purification. This protein was purified by the same method as described previously (33) but with only a single IMAC purification step followed by preparative size-exclusion chromatography on Superose-6 Increase (GE Healthcare) in a 16/60 column. For all final proteins, concentrations were determined by measuring sample absorbance at 280 nm (Nanodrop 2000C Spectrophotometer, Thermo Fisher Scientific) and using the extinction coefficient calculated from the amino acid sequence.

### Size-exclusion chromatography and SEC-MALS

For analytical SEC, freshly purified or thawed protein was centrifuged at 9300 × *g* for 10 min at room temperature in a benchtop centrifuge. Superose-6 Increase (GE Healthcare) or Superdex S-200 (GE Healthcare) in 10/300 GL columns were used on NGC^TM^ chromatography systems. Columns were equilibrated with final buffers (Table S2); 0.5 ml of protein sample was loaded, and the column was run at a flow rate of 0.5 ml/min. For SEC-MALS, proteins were centrifuged at 9300 × *g* for 10 min at room temperature. Superose-6 increase or Superdex S-200 in 10/300 GL columns were used on an Agilent 1200 LC system in-line with Wyatt Heleos (light scattering) and Wyatt Optilab T-Rex (refractive index) detectors to assess the molecular weight of the proteins. BSA (Thermo Fisher Scientific) was used as a standard. Astra software (Wyatt Technology, version 7.1.3) was used to collect and analyze the SEC-MALS data. Detailed information on buffers, injection rates, and flow rates can be found in Table S3.

### Differential scanning fluorimetry (thermal melting) analysis

Melting curves of purified full-length, ABC, DEF, and ABC + DEF complexes were obtained in triplicate on a Bio-Rad C1000 Thermo Cycler, and data were analyzed by Bio-Rad CFX Manager software (version 3.1). Proteins were thawed and centrifuged at 9300 × *g* for 10 min at room temperature in a benchtop centrifuge. A 50-μl reaction was prepared by mixing proteins at a final concentration of 1 mg/ml with 5 μl of 50× SYPRO Orange dye (Thermo Fisher Scientific) in the relevant final protein buffer (Table S2). Samples were heated to 25 °C for 10 min and then melt curves were measured from 25 to 95 °C with an increment of 0.2 °C per 0.01 min.

### Sedimentation velocity analytical ultracentrifugation

Sedimentation velocity experiments were carried out at 30,000 rpm at 20 °C on a Beckman Coulter ProteomeLab XL-I analytical ultracentrifuge and An50-Ti rotor following standard protocols (34). Samples of full-length neurofibromin at various concentrations (0.05–2.2 μm) in 20 mm HEPES, pH 7.4, 300 mm NaCl, and 1 mm TCEP were loaded in 12-mm two-channel Epon centerpiece cells. Sedimentation velocity data were collected using the absorbance (280 or 230 nm depending on the concentration) and interference optical detection systems. Time-corrected (35) data were analyzed in SEDFIT 16.1 (36) in terms of a continuous *c*(*s*) distribution of sedimenting species using an *s* range of 0–30 with a linear resolution of 300 and a maximum entropy regularization confidence interval of 0.68. The solution density and viscosity were measured experimentally at 20 °C on an Anton Paar DMA5000 density meter and Anton Paar AMVn viscometer, respectively. Protein partial specific volumes were calculated based on their composition in SEDNTERP (37). Samples of ABCD and CDEF domains were similarly studied at various loading concentrations at 40,000 rpm and 20 °C. ABCD was studied in 500 mm NaCl, 20 mm HEPES-NaOH, pH 7.3, and 5 mm TCEP, whereas CDEF was studied in 300 mm NaCl, 20 mm HEPES-NaOH, pH 7.3, and 5 mm TCEP. Similarly, samples of ABC and DEF were studied at 50,000 rpm at 20 °C in a solution buffer of 300 mm NaCl, 20 mm Tris-HCl, pH 8.5, and 5 mm TCEP. In all cases, data were analyzed in SEDFIT 16.1 as described above using a sedimentation coefficient resolution of 0.1 S and regularization confidence interval of 0.68. Equimolar mixtures of ABCD and EF domains, as well as ABC and DEF domains, were studied in 300 mm NaCl, 20 mm Tris-HCl, pH 8.5, and 5 mm TCEP at 50,000 rpm at 20 °C. Samples in the 1–5 μm concentration range were mixed from stock solutions of the individual domains just before the experiment, and data were analyzed as described above.

### Sedimentation equilibrium analytical ultracentrifugation

Sedimentation equilibrium experiments were carried out to confirm the oligomeric state of full-length neurofibromin. Samples in 20 mm HEPES, pH 7.4, 300 mm NaCl, and 1 mm TCEP were loaded at concentrations of 0.55, 1.1, and 2.2 μm in 12-mm six-channel Epon centerpiece cells (130 μl). Sedimentation equilibrium experiments were conducted at 20 °C and at 3000, 5000, and 7000 rpm on a Beckman Optima XL-A analytical ultracentrifuge and An50-Ti rotor following standard protocols (34), with absorbance data collected at 280 nm. Data were analyzed globally in terms of a single noninteracting species in SEDPHAT 14.0 to obtain the molar mass.

### Negative stain transmission EM

Freshly purified (full-length) or thawed (domains) proteins were centrifuged at 9300 × *g* for 10 min at 4 °C and diluted to 0.01 mg/ml in 20 mm Tris-Cl, pH 8.0, 300 mm NaCl, and 5 mm TCEP. Diluted protein was adsorbed on freshly glow-discharged carbon-film grid (CF200-CU, Electron Microscopy Sciences), washed with dilution buffer, and stained with 0.75% w/v uranyl formate, pH 4.5, for 30 s. Images of full-length neurofibromin were collected at ×67,000 magnification with EPU software from Thermo Fisher Scientific on a Tecnai T12 electron microscope equipped with a Gatan Ultra Scan camera, with images recorded at a pixel size of 1.77 Å. Images of CDEF were collected at ×100,000 magnification with SerialEM on a Tecnai T20 electron microscope equipped with an Eagle CCD camera, with images recorded at a pixel size of 2.19 Å. Images of ABC, ABCD, and DEF domains were collected at ×40,000 magnification on a Hitachi 7650 electron microscope operating at 80 kV with an AMT digital camera (Advanced Microscopy Techniques).

### 2D classification and 3D reconstruction of neurofibromin proteins

Negatively-stained neurofibromin particles were autopicked using EMAN2 e2boxer using a box size of 326 pixels (38). Reference-free 2D classification was carried out using the Iterative Stable Alignment and Clustering (ISAC) procedure implemented in a Sphire package (39). 41,951 particles were initially selected. Particles representing disordered NF1 2D class averages were separated, and the remaining particles were reclassified using ISAC. The final 294 class averages representing 30,588 particles were used for *ab initio* model reconstruction. This model was used as a reference for 3D refinement using meridian with C_2_ symmetry in Sphire. The final resolution of the reconstructed model was ∼20 Å, as estimated by Fourier shell correlation (criterion of 0.5). All further analyses and visualizations were performed using Chimera (40). The 3D variability analysis was done on the final refined model in Sphire. The final model data were submitted to wwPDB under the accession code EMD-20667. Approximately 28,800 particles of the NF1-CDEF domain were auto-picked using EMAN e2boxer, and 2D classification was performed with ISAC procedure using the same approach as described above. Particles of complexes of NF1-ABC and NF1-DEF were analyzed in a similar fashion with initial 2D classification using Sphire. Full-length NF1 dimer data were low-pass filtered to 60 Å and used as a reference in Relion for refinement of the model. 384 particles of the complex were used for the final reconstruction.

### Small-angle X-ray and neutron scattering data collection and analysis

SAXS experiments were performed on a Rigaku BioSAXS-2000 equipped with a Pilatus 100 K detector (Rigaku Americas, The Woodlands, TX). A fixed sample–to–detector distance, calibrated with a silver behenate standard, was used to collect scattering profiles of neurofibromin (0.5 and 1.0 mg/ml) at 20 °C. The data were reduced with the instrument software to obtain the scattering intensity, *I*(*Q*), *versus* wave vector transfer, *Q* (= 4π sin(θ)/λ, where 2θ is the scattering angle), and then buffer background was subtracted.

SANS experiments were performed on the Bio-SANS (CG-3) beamline at the High Flux Isotope Reactor located at Oak Ridge National Laboratory. Measurements were made using a 15.5-m sample–to–detector distance for the main 1 × 1-m detector and 6 Å wavelength neutrons (41). The wing detector bank was utilized to capture scattering at high angles. Neurofibromin samples in H_2_O buffer (1 mg/ml) were loaded into 1-mm pathlength circular-shaped quartz cuvettes (Hellma USA, Plainville, NY), and measurements were performed at 20 °C. Data reduction followed standard procedures using MantidPlot (42). The measured scattering intensity was corrected for the detector sensitivity and scattering contribution from the solvent and empty cells and were then placed on an absolute scale using a calibrated standard (43).

The reduced SAXS and SANS data were analyzed using the ATSAS tools (44) PRIMUS and GNOM for Guinier and *P*(*r*) analysis, respectively. Reported zero-angle scattering intensity, *I*(0), and radius of gyration, *R_g_*, values were obtained from the *P*(*r*) analysis. The neurofibromin molecular mass was calculated from *I*(0) and *R_g_* of the SAXS data using the method of Ref. 45. The neurofibromin mass was determined from *I*(0) of the SANS data as shown in Equation 1,

(Eq. 1) $$M=I\left(0\right)N_{A}/\left(c{\left(\Delta \rho \right)}^{2}{\bar{v}}^{2}\right)$$

where *N*_A_ = Avogadro's number; *c* = protein concentration (= 1 mg/ml); Δρ = scattering length density contrast between protein and buffer solution (= ρ_prot_ − ρ_buf_); and *v̄* = protein partial specific volume (= 0.74 ml/g). Using the contrast module of MULCh (46), the NF1 sequence was used to calculate Δρ in H_2_O buffer (= 2.364 × 10^10^ cm^−2^). The DAMMIF program within ATSAS was used to generate *ab initio* shape reconstruction models to fit the neurofibromin (0.5 mg/ml) SAXS data. A total of 20 models were generated and compared by the program. A theoretical SAXS curve was generated from the final negatively-stained EM model of neurofibromin holoprotein using the ATSAS tool EM2DAM.

### Immunoprecipitation and Western blotting

An NF1-null HEK293T cell line was generated using CRISPR to knock out both alleles of *NF1*. HEK293T and NF1-null 293T cells were maintained in DMEM with 10% fetal bovine serum. Transfections of plasmids into cells were performed using FuGENE 6 (Promega) according to the manufacturer's protocol, and the cells were harvested 24 h after transfection. Cells were lysed in a lysis buffer containing 20 mm Tris-Cl, pH 7.5, 150 mm NaCl, 5 mm MgCl_2_, 1% Triton X-100, and protease inhibitor mixture (Sigma). Immunoprecipitation was performed using EZview Red Anti-FLAG M2 Affinity Gel (Sigma). Beads were incubated with the cell lysates for 2 h at 4 °C and washed three times with cold lysis buffer. Primary antibodies (Cell Signaling Technology) used for Western blotting were FLAG (rabbit, 14793), V5 (rabbit, 13202), and HA (rabbit, 3724). NIR secondary antibodies (LI-COR Biosciences) were used and images were obtained using a LI-COR Odyssey scanner.

### Measurement of GAP activity in cells using a RAS–GTP ELISA

The NF1-null HEK293T cell line was transfected with a 3×FLAG-tagged KRAS4b plasmid along with a series of NF1 domain plasmids using FuGENE 6 (Promega) and grown in serum-free DMEM for 18 h. Cells were stimulated with 100 ng/ml EGF for 5 min and harvested per manufacturer's instructions for the RAS GTPase Chemi ELISA kit (Active Motif). Lysates were assayed by BCA protein assay prior to the ELISA. The Ras GTPase Chemi ELISA was performed as per the manufacturer's protocol with one exception: the second incubation (after samples and controls were added to the wells) was done at 4 °C instead of room temperature. For samples, 100 μg of cell lysate was added to each well, whereas control wells were filled with 50 μl of Lysis/Binding Buffer. The plate was read in a PerkinElmer Life Sciences Envision plate reader and imaged in a Bio-Rad ChemiDoc within 15 min of adding chemiluminescent reagents. Western blots to verify protein expression levels were run on Tris acetate or Bolt gels (30 μg of cell lysate/lane) and probed with a series of antibodies: pEGFR (Cell Signaling 3777), vinculin (Cell Signaling 19301), HA-tag (Cell Signaling 2367), V5-tag (Cell Signaling 1320S), FLAG-tag (Sigma, F1804), KRAS (Sigma/Novus Biologicals WH0003845M1/H0003845M1), and Li-Cor Secondary Antibodies (IRDye® secondary antibodies 800CW and 680RD).

### Phosphate sensor GAP-stimulated GTP hydrolysis assay

Assays for GAP-stimulated GTP hydrolysis were set up in 50 mm Tris-HCl, pH 7.5, 150 mm NaCl, 1 mm TCEP, and 1 mm MgCl_2_ with a final concentration of 2.5 μm phosphate sensor protein (Thermo Fisher Scientific) and 20 μm GTP and kept on ice until the assay was started. NF1 proteins were diluted in the same buffer and added to the assay along with controls containing no NF1 proteins or added phosphate. A 96-well black flat-bottom plate (Corning catalog no. 3544) was used in the assay, with 100 μl total volume per well. Reactions were initiated by addition of 3 μm KRAS4b(1–169) protein, and readings were taken every 10 s in a BMG Fluostar Omega plate reader with an excitation wavelength of 430 nm and an emission wavelength of 460 nm. Initial rates of activity were calculated from the linear portion of the data (*R*^2^ >0.95) and compared with the intrinsic hydrolysis rate in the absence of added NF1 proteins.

## Author contributions

M. S., S.-W. H., S. M., S. S., D. V. N., W. G., F. M., and D. E. conceptualization; M. S., S.-W. H., R. G., S. M., M. D., P. J., C. B. S., D. B., S. S., F. M., and D. E. resources; M. S., W. G., and D. E. data curation; M. S., S.-W. H., R. G., S. M., M. D., P. J., H. O., C. B. S., W. G., and D. E. validation; M. S., S.-W. H., R. G., M. D., D. R., T. W., P. J., C. B. S., and D. B. investigation; M. S., S.-W. H., R. G., P. J., H. O., C. B. S., D. B., W. G., and D. E. visualization; M. S., S.-W. H., R. G., S. M., M. D., P. J., H. O., C. B. S., S. S., W. G., and D. E. methodology; M. S., R. G., S. M., P. J., H. O., A. R., W. G., F. M., and D. E. project administration; R. G., H. O., C. B. S., and D. B. formal analysis; R. G., H. O., A. R., D. V. N., F. M., and D. E. funding acquisition; R. G., H. O., C. B. S., W. G., and D. E. writing-original draft; S. M., H. O., A. R., S. S., D. V. N., W. G., F. M., and D. E. supervision.

## Supplementary Material

Supporting Information

## Footnotes

5 W. Yan, personal communication.

6 W. Yan and D. Simanshu, submitted for publication.

## Footnotes

4 The abbreviations used are:

GAP: GTPase-activating protein
NF1: neurofibromatosis type 1
GRD: GAP-related domain
PH: pleckstrin homology
SEC: size-exclusion chromatography
SEC-MALS: size-exclusion chromatography multiangle light scattering
SAXS: small-angle X-ray scattering
SANS: small-angle neutron scattering
IP: immunoprecipitation
MBP: maltose-binding protein
TEV: tobacco etch virus
EGF: epidermal growth factor
IMAC: immobilized metal-affinity chromatography
WCL: whole-cell lysate
TBD: tubulin-binding domain
Sec-PH: Sec14-pleckstrin homology
HEAT: HEAT-like repeat domain
DMEM: for Dulbecco's modified Eagle's medium
TCEP: tris(2-carboxyethyl)phosphine
mTOR: mechanistic target of rapamycin.
