# Supplementary material for: Biochemical and structural analyses reveal that the tumor suppressor neurofibromin (NF1) forms a high-affinity dimer
Source: J Biol Chem. 2019 Dec 13;295(4):1105–19. doi: 10.1074/jbc.RA119.010934 (PMC6983858; doi:10.1074/jbc.RA119.010934)
Supplement: Supporting Information [file supp_295_4_1105__index.html]

Biochemical and structural analyses reveal that the tumor suppressor neurofibromin (NF1) forms a high-affinity dimer — Neurofibromin is a high-affinity dimer — Biochemical and structural analyses reveal that the tumor suppressor neurofibromin (NF1) forms a high-affinity dimer — Neurofibromin is a high-affinity dimer — Supporting Information 

# Biochemical and structural analyses reveal that the tumor suppressor neurofibromin (NF1) forms a high-affinity dimer

## Supporting Information

- Supporting Information (to be published online) - Supporting info (11 figures and 3 tables)
