# Supplementary material for: Biochemical and structural analyses reveal that the tumor suppressor neurofibromin (NF1) forms a high-affinity dimer
Source: J Biol Chem. 2019 Dec 13;295(4):1105–19. doi: 10.1074/jbc.RA119.010934 (PMC6983858; doi:10.1074/jbc.RA119.010934)
Supplement: Supporting Information [file supp_RA119.010934_155633_2_supp_443999_q2gtsf.pdf]

Biochemical and structural analyses reveal that the tumor suppressor neurofibromin (NF1) forms a high-affinity dimer

Mukul Sherekar*[1], Sae-Won Han*[2,3], Rodolfo Ghirlando[4], Simon Messing[1], Matthew Drew[1], Dana Rabara[1], Timothy Waybright[1], Puneet Juneja[5], Hugh O'Neill[6], Christopher B. Stanley[6], Debsindhu Bhowmik[6], Arvind Ramanathan[7], Sriram Subramaniam[8,9], Dwight V. Nissley[1], William Gillette[1], Frank McCormick[1,2], and Dominic Esposito[1†]

Supporting information includes 3 tables and 11 figures

Table S1: Neurofibromin protein size and mass predictions from SAXS and SANS analysis. Values are calculated from the data in Fig. 1D and Fig. S5, including radius of gyration (R_g), maximum dimension (D_{max}), predicted molecular mass (M) and predicted oligomeric state.

Protein	Experiment	I(0)[1]	R_g (Å)	D_{max} (Å)	M (kDa)	Oligomer (M/M_{mon})[2]
Full-length	SAXS (1.0 mg/ml)	9.99 ± 0.1	89.4 ± 0.9	270	670 ± 20	2.11 ± 0.06
Full-length	SAXS (0.5 mg/ml)	4.73 ± 0.08	88.0 ± 1.0	258	630 ± 20	1.99 ± 0.06
Full-length	SANS (1.0 mg/ml)	0.302 ± 0.004	107.8 ± 0.9	300	593 ± 8	1.87 ± 0.03
ABCD	SAXS (1.5 mg/ml)	6.2 ± 0.1	67.1 ± 0.7	205	201 ± 6	0.98 ± 0.03
CDEF	SAXS (4.3 mg/ml)	6.6 ± 0.1	73.5 ± 0.7	228	450 ± 10	2.05 ± 0.05

[1]SAXS I(0) values are in arbitrary units; SANS I(0) = [cm^{-1}].

[2]M_{mon}, monomer molecular weight; 317 kDa (full length), 220 kDa (CDEF), 206 kDa (ABCD)

Table S2: Buffers used in protein purification. Cell pellets were homogenized in the lysis buffers below prior to lysis. Lysis buffers also served as the base buffers for the IMAC purification steps. The final buffers were used as the running buffer during preparative size exclusion chromatography and for long-term storage of purified proteins. All lysis buffers also contained protease inhibitors (1:100 v/v, Sigma P-8849), and all final buffers contained protease inhibitors (1:1000 v/v, Sigma P-8849). Where two final buffers are noted, the first was used in the data shown in the manuscript, while the buffer in parentheses was used to confirm behavior of the protein in lower salt condition size exclusion chromatography.

Domain	Amino Acids	Lysis Buffer[1]	Final Buffer[1]
Full length	2-2818	A	A (G)
ABC	2-1540	B	C (G)
ABCD	2-1820	D	D
CDEF	861-2818	D	E
DEF	1541-2818	B	C (G)
EF	1821-2818	C	C
C*D	1085-1816	C	C
D	1541-1820	E	F
C*	1085-1530	D	F
GRD	1172-1530	E	F
GRD	1198-1530	E	F
GRD	1203-1530	E	F

[1]A: 20 mM Tris-Cl, pH 8.0, 300 mM NaCl, 5 mM TCEP
B: 20 mM Tris-Cl, pH 8.5, 500 mM NaCl, 10% (v/v) glycerol, 5 mM TCEP
C: 20 mM Tris-Cl, pH 8.5, 300 mM NaCl, 5 mM TCEP
D: 20 mM HEPES, pH 7.3, 500 mM NaCl, 5 mM TCEP
E: 20 mM HEPES, pH 7.3, 300 mM NaCl, 1 mM TCEP
F: 20 mM HEPES, pH 7.3, 150 mM NaCl, 1 mM TCEP
G: 20 mM Tris-Cl, pH 8.5, 150 mM NaCl, 1 mM TCEP

Table S3: SEC-MALS Buffers/Conditions. All running buffers contained 20 mM Tris-Cl (at the indicated pH), 300 mM NaCl, and 5 mM TCEP.

**Domain**	**SEC Resin**	**Running Buffer pH**	**Injection Volume (μl)**	**Protein Load (μg)**	**Flow Rate (ml/min)**
Full length	Superose-6 Increase	8.0	100	100	0.5
ABC	Superose-6 Increase	8.5	100	100	0.3
ABCD	Superdex S200	8.0	50	127	0.3
CDEF	Superdex S200	8.0	50	100	0.3
DEF	Superose-6 Increase	8.5	50	65	0.3
EF	Superose-6 Increase	8.5	100	80	0.3

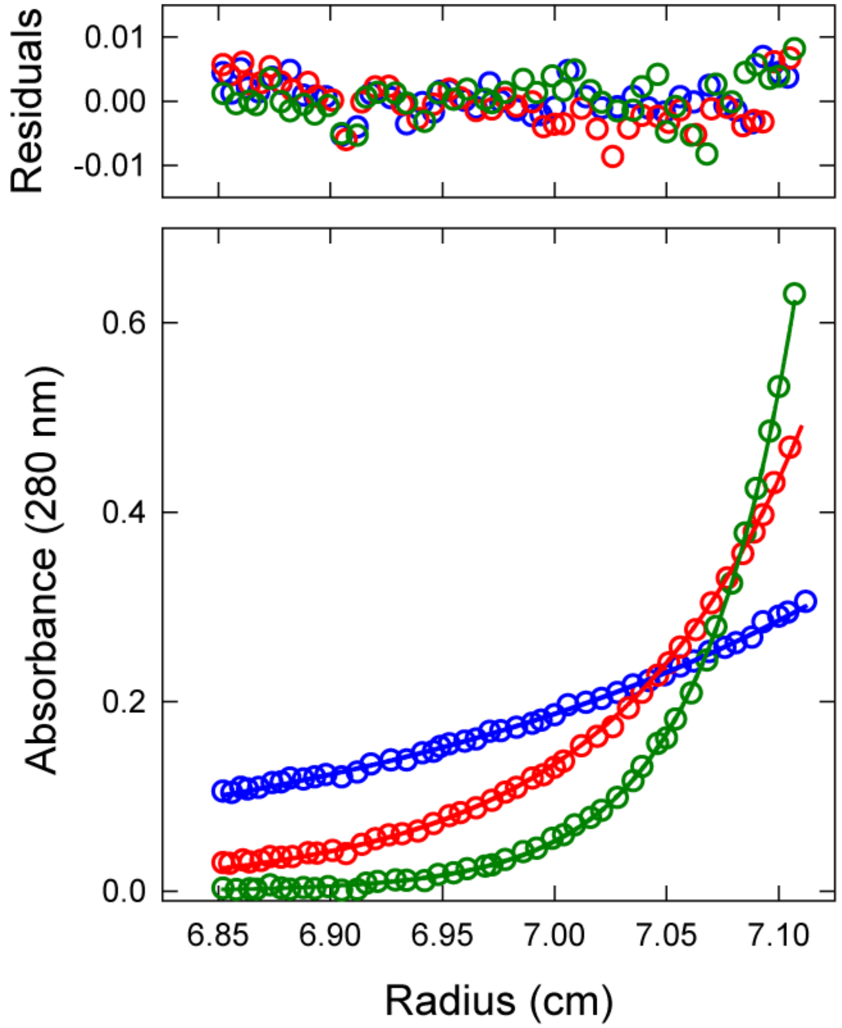

Figure S1: Sedimentation equilibrium of full-length neurofibromin. Sedimentation equilibrium absorbance data for a solution of neurofibromin at 550 nM collected at 3,000 (blue), 5,000 (red) and 7,000 (green) rpm analyzed in terms of a non-interacting single ideal solute. Solid lines show the global best-fit, with the corresponding residuals shown in the plot above. For clarity, every third experimental data point is displayed.

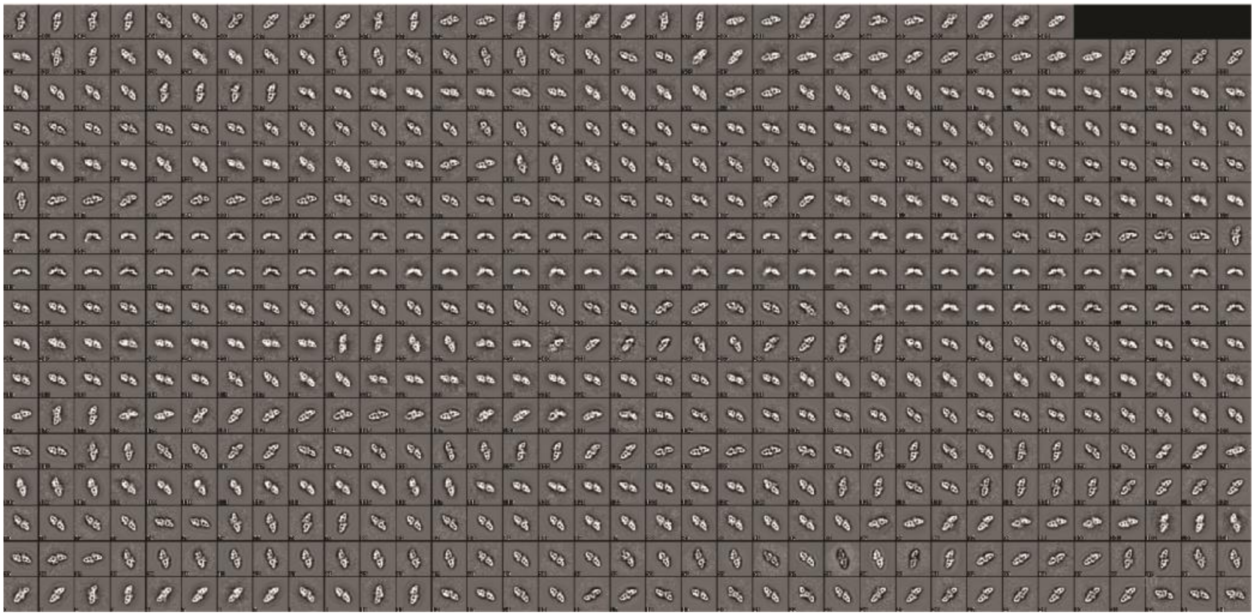

Figure S2: Back projection of neurofibromin EM model to 2D class averages. The 3D EM model generated was used to calculate 2D back projections using EMAN2. A representative sample of the back projected 2D classes is shown here. Each box is 50 nm x 50 nm in size.

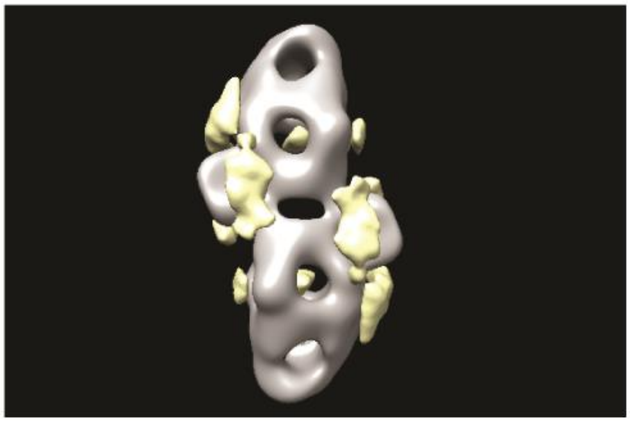

Figure S3: 3D variability analysis of neurofibromin structure. 3D variability analysis showing heterogeneity in the 3D model of full-length neurofibromin. The heterogenous areas are shown in yellow.

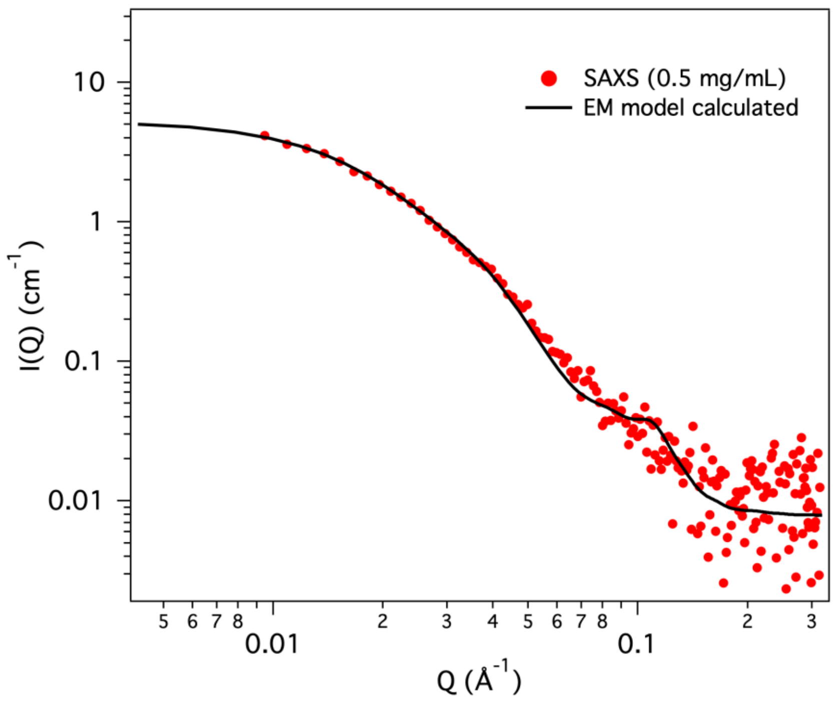

Figure S4: Prediction of neurofibromin SAXS behavior using the EM model. SAXS data of full-length neurofibromin is shown in red, while the black line represents theoretical SAXS data predicted by the 3D EM model in Fig. 2.

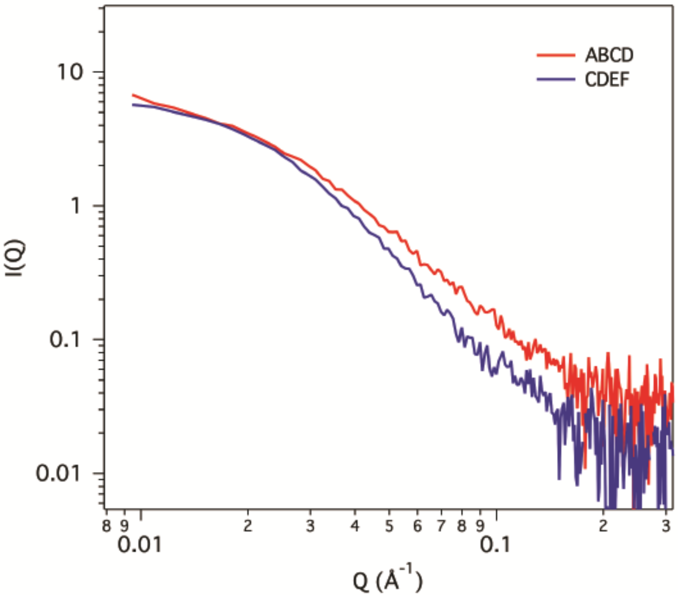

Figure S5: Small-angle X-ray scattering of ABCD and CDEF fragments of neurofibromin. ABCD protein (red line) was measured at 1.5 mg/ml (7.5 uM) and CDEF protein (blue line) was measured at 4.3 mg/ml (16 uM).

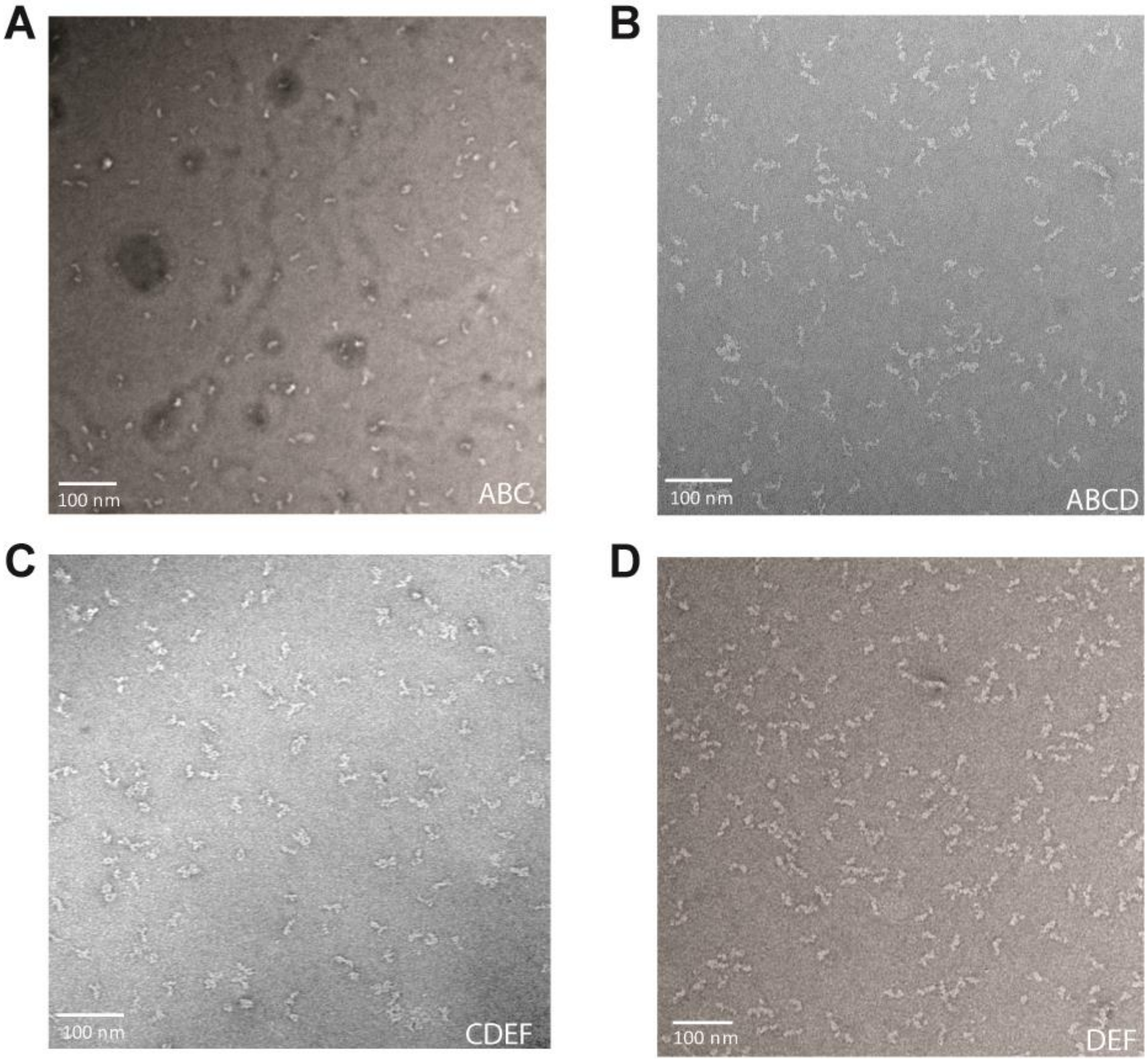

Figure S6: Negative stain transmission EM images of neurofibromin domain proteins. Representative transmission electron micrographs of purified ABC (panel A), ABCD (panel B), CDEF (panel C), and DEF (panel D) proteins.

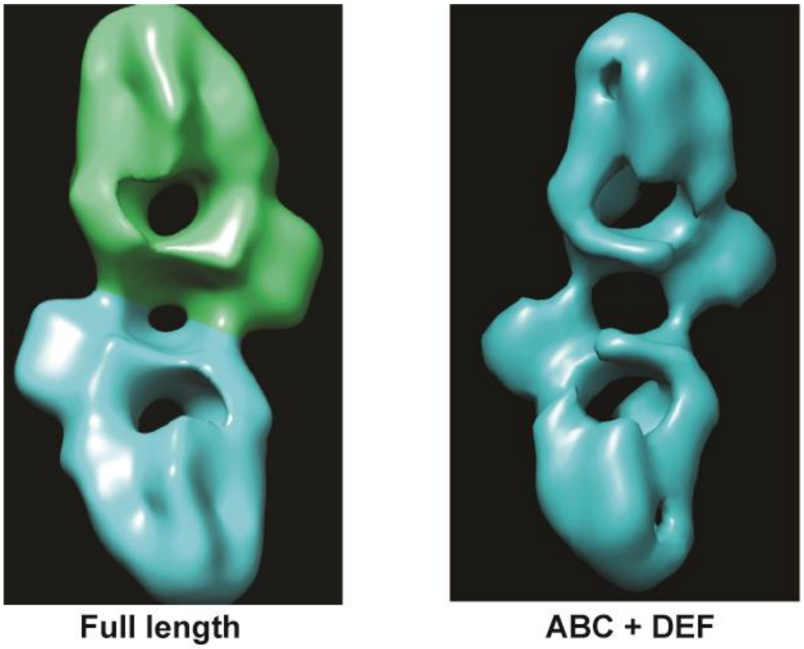

Figure S7: Model comparison of full-length neurofibromin dimer and reconstituted ABC+DEF dimer. The model from Figure 2C is compared to a model generated from the SEC-purified ABC+DEF complex. Only 384 particles were used for the reconstruction of the complex, leading to the lower resolution.

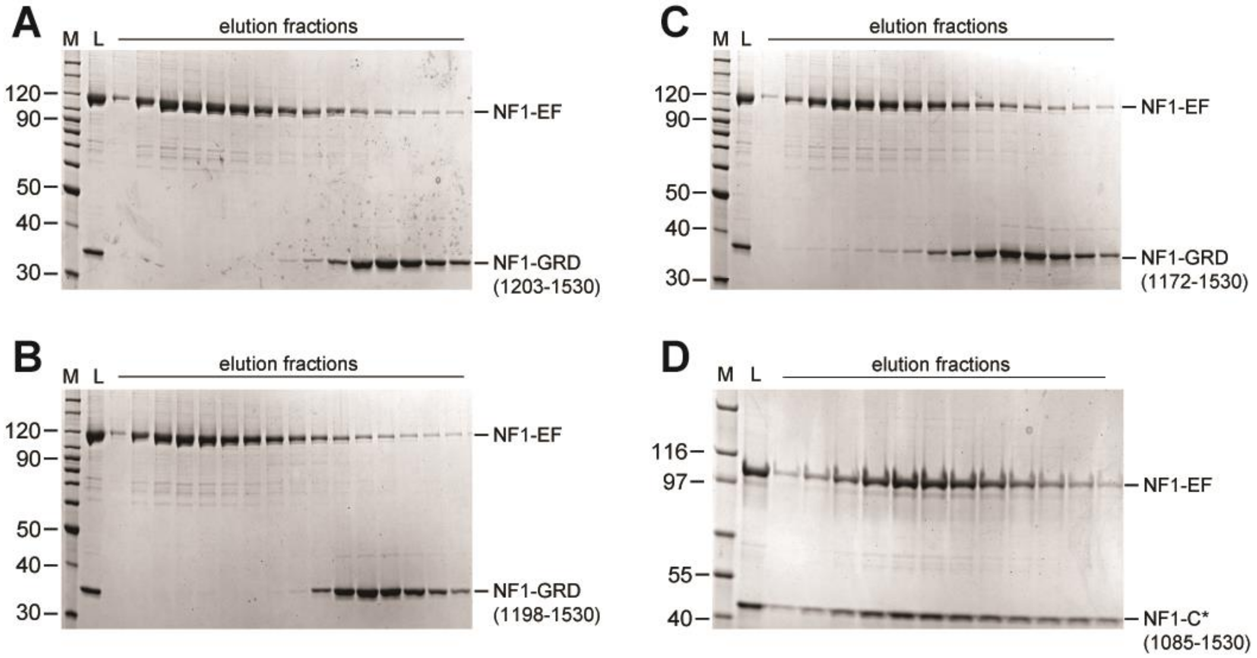

Figure S8: Formation of complexes of neurofibromin C and EF domains requires the presence of the TBD region. SDS-PAGE analysis of SEC of equimolar mixtures of NF1 domains with EF (1821-2818). In each panel, the size of molecular weight markers in lane M is noted in kilodaltons. Lane L represents the loaded material, and additional lanes are elution fractions across the column. Neurofibromin fragments mixed with the EF domain in each panel were: *A*, 1203-1530; *B*, 1198-1530; *C*, 1172-1530; *D*, 1085-1530. Coeluting proteins are observed only in the construct containing amino acids 1085-1171.

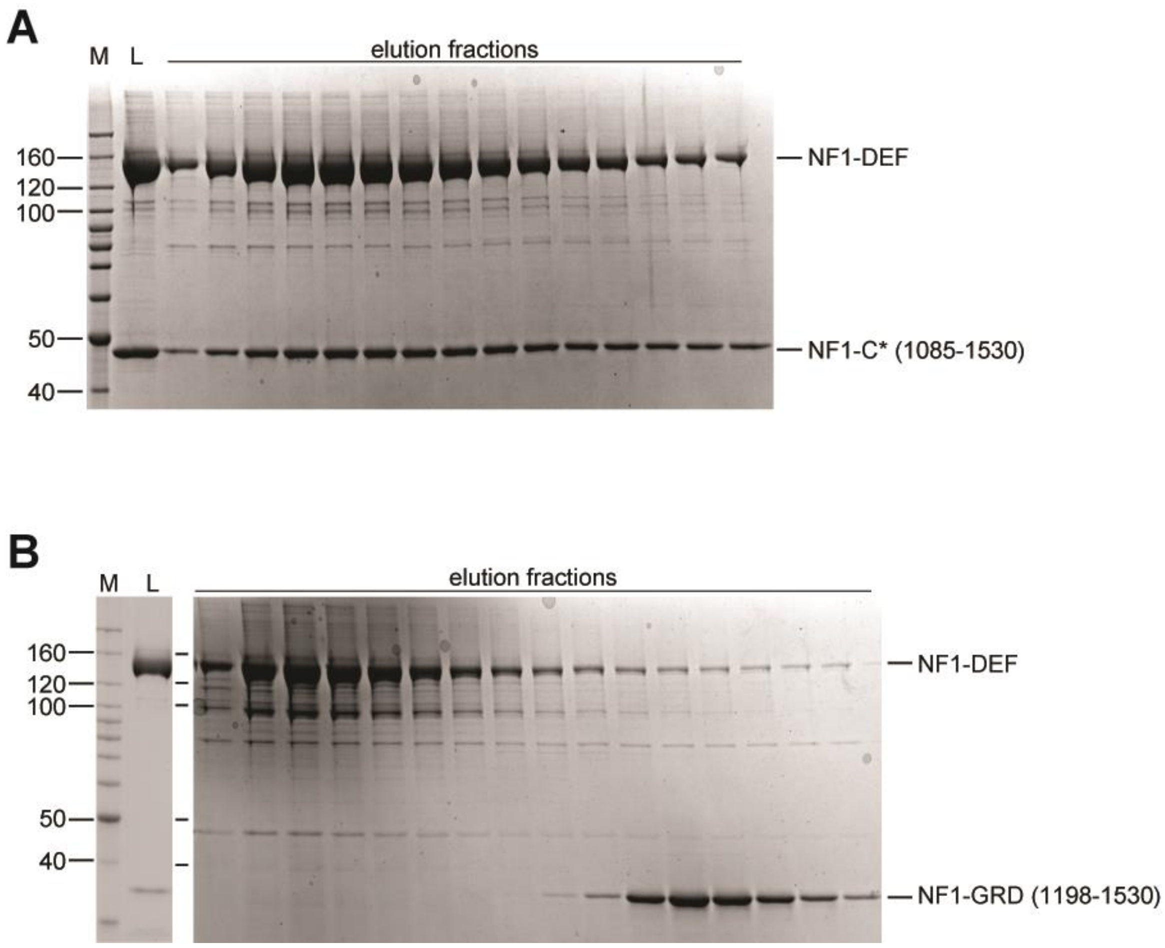

Figure S9: Formation of complexes of neurofibromin C and DEF domains requires the presence of the TBD region. SDS-PAGE analysis of SEC of equimolar mixtures of NF1 domains with DEF (1541-2818). In each panel, the size of molecular weight markers in lane M is noted in kilodaltons. Lane L represents the loaded material, and additional lanes are elution fractions across the column. Neurofibromin fragments mixed with the DEF domain in each panel were: *A*, 1085-1530; *B*, 1198-1530. Coeluting proteins are observed only in the construct containing amino acids 1085-1197.

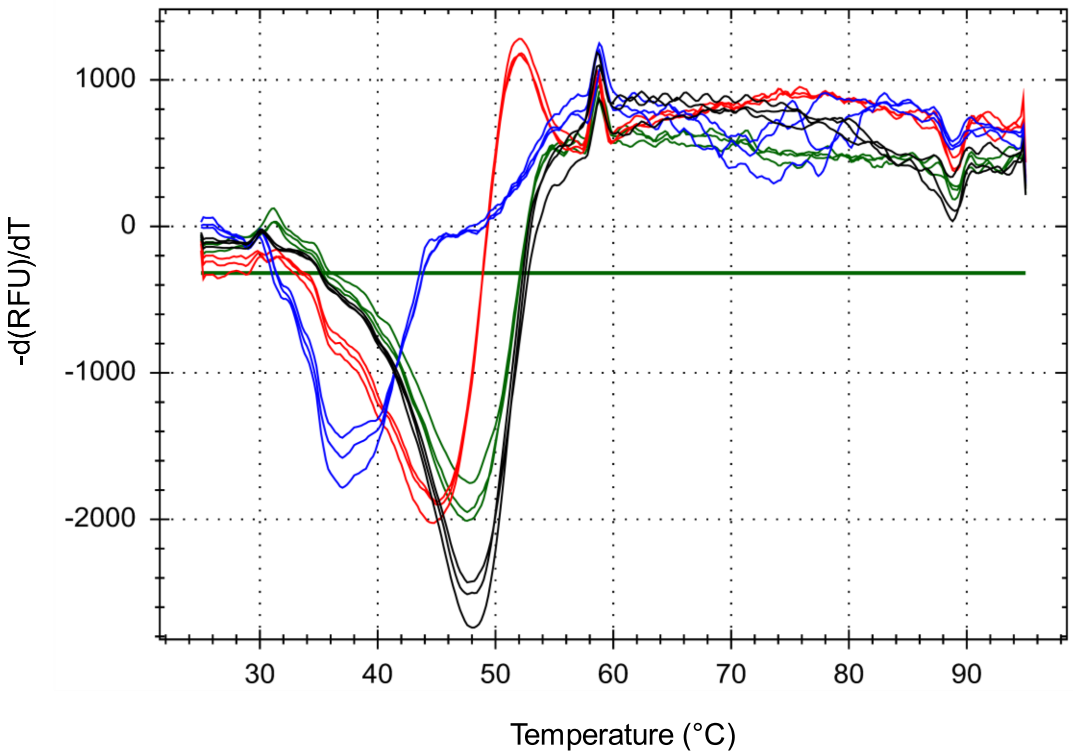

Figure S10: Differential scanning fluorimetry analysis of neurofibromin proteins. Thermal melt curves of triplicate measurements of neurofibromin proteins: full-length (black), ABC (blue), DEF (red), and reconstituted ABC+DEF (green). Calculated Tm values for the proteins are 47.7 °C (full-length), 47.6 °C (ABC+DEF), 45 °C (DEF), and 37 °C (ABC).

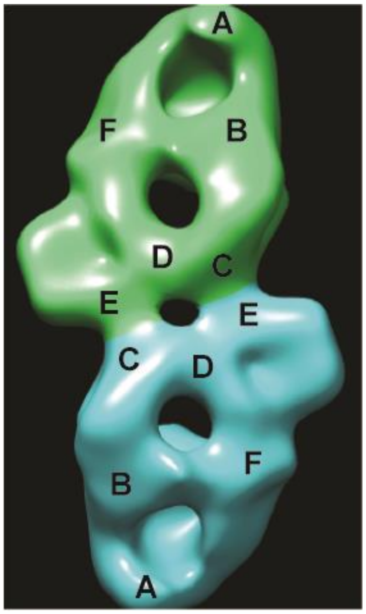

Figure S11: Potential model for the proposed interaction of neurofibromin C and E domains. One possible model for how the subunits of neurofibromin are aligned in the dimer which is consistent with experimental observations and the size of the various domains.
